# Randomised Controlled Trial for the Evaluation of the Efficacy of the IDA’s “Living Well” Online Counselling Tool in First-Time Adult Users with Hearing Loss

**DOI:** 10.3390/audiolres14050071

**Published:** 2024-09-19

**Authors:** Evgenia Vassou, Eleftheria Iliadou, Nikolaos Markatos, Dimitrios Kikidis, Athanasios Bibas

**Affiliations:** 1st University Department of Otolaryngology and Head & Neck Surgery, National and Kapodistrian University of Athens, 11527 Athens, Greece; iliadoue@med.uoa.gr (E.I.); markatosn@med.uoa.gr (N.M.); dimi-triskikidis@yahoo.com (D.K.); ampimpas@med.uoa.gr (A.B.)

**Keywords:** hearing aids, counselling, living well, coping strategies, communication, communication profile for the hearing impaired, hearing handicap inventory

## Abstract

Effective management of hearing loss through the use of modern hearing aids significantly improves communication and the quality of life for individuals experiencing auditory impairment. Complementary counselling of patients with hearing loss who will be fitted with hearing aids for the first time should be evidence-based and adapted to their individual needs. To date, several counselling protocols and tools have been developed. The aim of this randomised controlled trial study was to investigate the efficacy of the application of the IDA’s “Living Well” counselling tool in first-time hearing aid users in terms of the degree of their hearing related handicap (using the Hearing Handicap Inventory (HHI)), their communication coping strategies (using the Communication Profile for the Hearing Impaired (CPHI)) and their overall satisfaction of the hearing aids (using a Likert scale). Both groups (the IDA and the control group) were fitted with hearing aids and received counselling for their hearing aids by the same audiologist. The IDA group attended an additional counselling session about communication coping strategies with the use of the “Living Well” tool. Both groups’ participants were seen for their hearing aid fittings 4–6 weeks, 3 and 6 months after their fitting when the HHI and the CPHI were measured. Although there was not a statistically significant difference between the two groups for the primary and secondary outcomes, the IDA group did show a more consistent improvement of their HHI score and less frequent use of maladaptive strategies. The “Living Well” counselling tool proved to be a favourably received and helpful counselling tool in first-time hearing aid users.

## 1. Introduction

According to the WHO’s estimations, 6% of the global population (466 million people) are affected by handicapping hearing loss [1,2]. By 2050, hearing loss will affect more than 2.5 billion of people. That means practically 1 out of 4 people, over 700 million people, will be in need of hearing rehabilitation [1,2]. The annual cost for Europe is estimated to be 213 billion euros per year, while globally, the cost reaches 750 billion dollars [1,2]. Apart from the financial cost, hearing loss is related to significant societal and personal burden and should not be considered as an isolated health problem [3]. Multiple studies imply association of hearing impairment with psychosocial and physical diseases such as cognitive disorders and dementia, anxiety and depression, accidents and higher mortality rates [4,5,6,7]. People who suffer from hearing loss are at risk of developing a declining social life and social interactions, distant interpersonal relationships, physical and emotional health issues and low vocational ambitions [8]. Moreover, people suffering from hearing loss are less active and tend to retire sooner than normal-hearing people [9]. Elderly people with hearing impairment tend to isolate themselves by eliminating any participation in social events [5,6,7]. The lack of communication with their environment significantly lowers their quality of life. It has been shown that the communication handicap is often identified as the most crucial effect of hearing impairment [10].

Although the only currently available and validated management solution for hearing loss is the fitting and use of hearing assistive devices, only 40% of the estimated 430 million people needing one will seek, acquire and continue using it [11,12]. This phenomenon is partly attributed to patients’ limited access to healthcare, suboptimal education about their hearing loss or non-personalised approach to fitting and counselling that fails to cover their needs. Several studies reported that overall pre-fitting expectations about hearing aid performance are high among non-users of hearing aids [13,14]. 

Key points to the satisfaction of hearing aid users and thus the minimisation of drop-out risk are the adequate fitting of the device, the affordability and accessibility of the follow-up services (65% of individuals with hearing loss would search for treatment if they could pay for the service) and their proper combination with thorough and evidence-based personalised counselling [4,15]. In order to efficiently counsel patients with hearing loss, physicians should take into account their comorbidities and degree of handicap, their preferences and needs and their daily challenges (personal or environmental). This personalised approach should not omit the coping strategies that people with hearing loss use in their effort to improve their communication. These coping strategies can be divided into adaptive and maladaptive strategies: Adaptive strategies, such as asking others to repeat their words, are behaviours that presumably improve communication. Maladaptive strategies, such as pretending to understand the conversation when they are not, are behaviours that do not promote communication and can cause further frustration and social isolation [16,17,18]. 

Several counselling programs have been developed through the years. In our study we have used the IDA’s “Living Well” counselling tool which was developed by the IDA Institute in 2011 and is a free online tool (www.idainstitute.com/livingwell (accessed on 20 August 2024)). The institute’s website provides professionals with free online instructional videos on how to use the counselling tool. The IDA’s “Living Well” tool uses photographs of everyday situations as a starting point for a conversation with the patients about the difficulties they may face in similar situations due to their hearing impairment. Taking into account the information reported by the patients, the counsellor is able to develop an individualised and focused therapy plan with specific communication and technological strategies tailored to them and their real everyday life routines [19]. To date, there is no study assessing the efficacy of the IDA’s “Living Well” tool as a complementary counselling tool in first-time hearing aid users.

This is a mixed-methods study that primarily aimed to assess qualitatively and quantitively the efficacy of the IDA’s online “Living Well” counselling tool in the context of a Greek public audiology clinic setting. The aim of this study was to examine the effect of the implementation of the IDA’s “Living Well” tool in first-time hearing aid ambulatory adult non-deaf users with the development of adaptive communication coping strategies and the improvement of quality in their everyday communication and life. We hypothesised that the HHI/A-E score at 6 months for the IDA group would be significantly different than the control group (Primary Hypothesis or Hypothesis 1).

## 2. Materials and Methods

### 2.1. Study Design

This study was a 2-arm parallel randomised controlled trial.

### 2.2. Participants

Participants with the following inclusion criteria were recruited in the study:

1. Adults above 18 years old;
2. With Pure Tone Audiometry (PTA) thresholds (250 Hz–8000 Hz) between 40 dB HL and 90 dB HL sensorineural hearing loss and 50% of the correct repetition in Words in Babble (WiB) at +3 SNR [20,21];
3. Who agreed to be fitted for the first time with hearing aids;
4. Have a Montreal Cognitive Assessment (MOCA) score higher than 18.

The COVID-19 pandemic resulted in a decreased incoming number of patients in the hospital; thus, in April 2022, the eligibility criteria of the participants were changed and adults with Pure Tone Audiometry (PTA) thresholds (250 Hz–8000 Hz) between 30 dB HL and 120 dB HL sensorineural hearing loss, conductive and mixed hearing loss were also included. 

During their initial assessment, candidates were screened for their eligibility for the study. Those who fulfilled the inclusion criteria and were willing to participate, read, understood and signed the informed consent form (ICF). They then underwent a thorough structured medical and hearing history interview, otomicroscopy, tympanometry, standard pure tone audiometry (250–8000 Hz, including 3000 and 6000 Hz), speech audiometry, Uncomfortable Loudness Level (ULL) (if needed) and speech in noise audiometry. For the speech in noise audiometry, the Words-in-Babble in Greek tool was used [22]. The tests were conducted with the Affinity 2.0 audiometer (EN 60645-1, ANSI S3.6) using TDH39 headphones.

### 2.3. Interventions

Participants in the IDA group were evaluated four times. At their entry to the study, they received their hearing aids, counselling for the hearing aids (detailed counselling about use, care and maintenance of the hearing aids and adaptation to them) and counselling about potentially beneficial communication coping strategies with the help of the IDA’s “Living Well” counselling tool. They were then seen again 4–6 weeks later to repeat the face-to-face fine tuning of the hearing aids and the communication coping strategies counselling session, including a new interview (completion of the Hearing Handicap Inventory (HHI), the Communication Profile for the Hearing Impaired (CPHI) and a Likert scale score for the effectiveness of the “Living Well” tool and the satisfaction of the hearing aid usage). Online follow-up sessions were conducted at 3 and 6 months for further support of the participants if needed and for data collection. Participants in the control group were fitted with hearing aids and monitored in the exact same manner and frequency as the IDA group with the difference that they received counselling for the hearing aids (detailed counselling about use, care and maintenance of the hearing aids and adaptation to them) but not the IDA “Living well” counselling tool. All of the participants were assessed, fitted and counselled by the same audiologist (first author). The hearing aids were Receiver in the Canal (RIC) and were fitted using the NAL-NL 2 fitting formula after Real Ear Measurement (REM). The domes were chosen to reach, as closely as possible, to the REM’s prescribed target [23].

### 2.4. Outcomes

#### 2.4.1. Primary Outcome

The primary outcome was the participants’ score in the Hearing Handicap Inventory for Adults or Elderly (HHI-A or -E): The HHI-A and -E are validated tools that quantify the self-reported emotional and social effects of hearing loss in patients’ everyday life [24]. They both consist of 25 items. The HHI-A is the modified version of the HHI-E for use with younger hearing-impaired adults [25].

#### 2.4.2. Secondary Outcomes

The Communication Profile for the Hearing Impaired (CPHI) was selected as a secondary outcome. The CPHI is a self-assessment inventory that consists of 145 statements divided into four areas: Communication Performance, Communication Environment, Communication Strategies, and Personal Adjustment [26]. The inventory assesses the effects of hearing loss and the degree of those effects in the lives of individuals with hearing loss [27]. 

Prior to this study there was no Greek version of the CPHI. The questionnaire was forward–backward translated to Greek by one Greek and one native English and Greek speaker with satisfactory results. 

Other secondary outcomes included hours of hearing aid usage per day as calculated through the data logging, the participants’ satisfaction of using the hearing aids through a Likert scale at 6 months and the IDA group’s participants’ satisfaction with the “Living Well” counselling tool at 4–6 weeks as recorded with a 5-point Likert scale score.

### 2.5. Sample Size

The sample size was calculated based on our primary hypothesis with the help of G*power software 3.1. The required sample size to achieve 80% power for detecting an effect of 0.8, at a significance criterion of α = 0.05 was Ν = 26. Assuming that there would be dropouts, an increase of the sample size by 10% was made and 30 participants were recruited in each group. The significance level was set to 0.05 (5%). If the *p*-value was found to be less than or equal to 0.05 (the significance level), we would conclude that the result was statistically significant.

### 2.6. Randomisation

The randomisation was carried out with the help of the sealed envelope website (https://www.sealedenvelope.com/ (accessed on 28 September 2021)), and a blocked randomisation with a 10-block size was used. The randomisation, enrolment and participants’ assignments to the different groups were executed by the same audiologist. 

### 2.7. Statistical Methods

A Mann-Whitney test was used for testing our primary hypothesis, as the scores of the HHI/A-E were not normally distributed after D’Agostino–Pearson, Kolmogorov–Smirnov and Shapiro–Wilk tests. In exploratory analyses, the Mann-Whitney test was used to compare the two groups for their HHI/A-E scores at 3 months, their CPHI score at 6 months, their average hearing aid usage per day and their overall satisfaction of using the hearing aids (Likert scale) at 6 months.

## 3. Results

### 3.1. Participants

**Figure 1:**
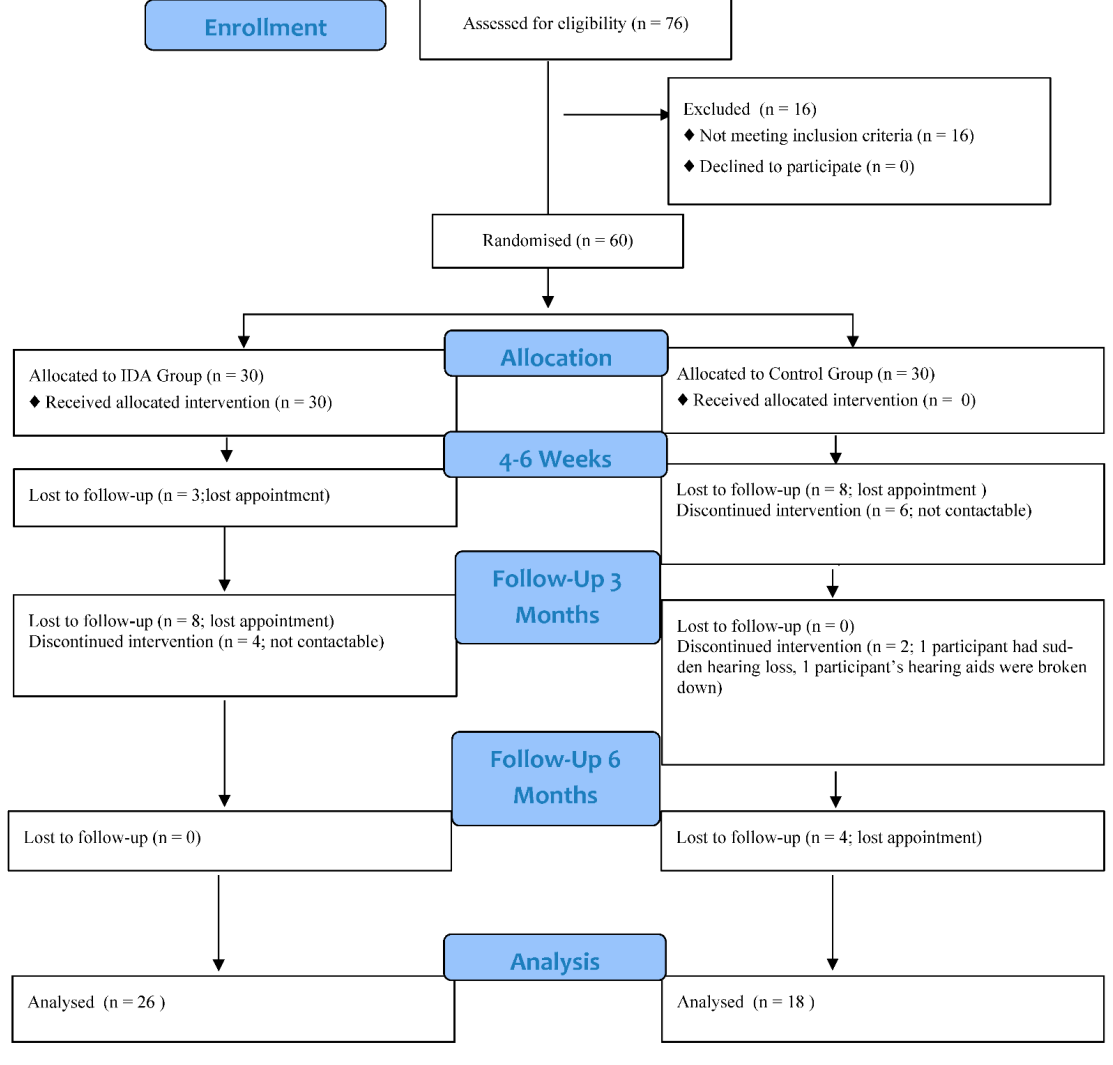
Consort flow diagram.

The recruitment started in September 2021 and lasted until August 2022 and the follow-up sessions took place 3 months and 6 months after each participant’s recruitment. A total of 76 candidates were screened, 60 of whom were finally recruited in the study. The flow diagram is shown in Figure 1 and the baseline data are shown in Table 1.

### 3.2. Primary Hypothesis

The IDA group scored lower (M = 24.92, SD = 20.84, Median = 18.00, Interquartile Range (IQR) = 20.50) than the control group (M = 29.67, SD = 20.49, Median = 18.00, IQR = 20.50) (Table 2) at 6 months after their entry to the study. Nevertheless, no statistically significant difference between the two groups was found (Mann–Whitney U = 189, *p* = 0.29, n1 = 26, n2 = 18).

### 3.3. Secondary Outcome Measures

#### 3.3.1. Communication Profile for the Hearing Impaired

**Figure 2:**
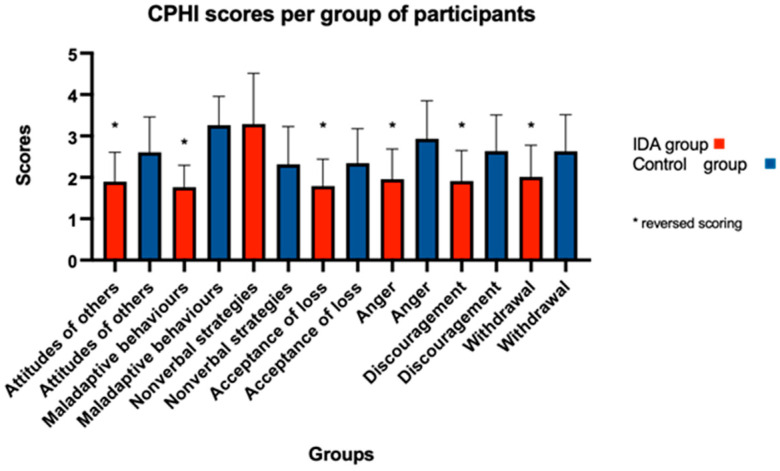
CPHI scores with IDA group’s higher scores.

**Figure 3:**
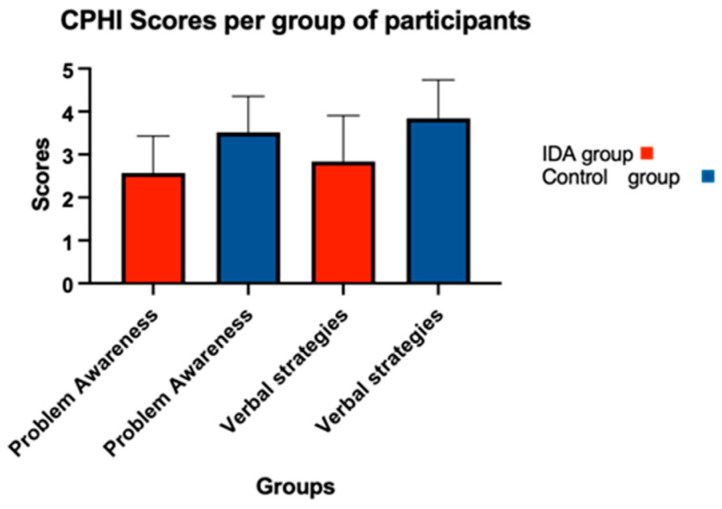
CPHI scores with control group’s higher scores.

All of the participants completed the CPHI questionnaire 6 months after their hearing aid fittings. The participants in the IDA group felt that they perceived fewer negative beliefs and reactions from others to their hearing loss and communication difficulties, and they reduced the maladaptive communication strategies they used to use (ignoring, interrupting others, pretending to understand and avoiding conversing with others). Furthermore, they better accepted their hearing impairment with fewer outbursts of anger and annoyance in response to communication obstacles. They also showed rarer feelings of discouragement and depression due to their hearing loss and communication problems and had a decreased tendency to withdraw from social interaction (Figure 2, Table 3). The participants in the control group showed higher awareness of common communication problems arising from hearing loss and were able to communicate their needs to others (Figure 3, Table 3).

Figure 2. The graph shows the IDA group’s mean and standard deviations, which are better than the control group’s. All these scales are reversed for scoring (the higher score indicates a higher level of difficulty). The bars show the mean, and the error bars show the standard deviation.

Figure 3. The graph shows the control group’s higher scores compared to the IDA group’s scores. The bars show the mean, and the error bars show the standard deviation.

#### 3.3.2. Hearing Handicap Inventory for Adults/Elderly (3 Months Completion)

At 3 months, the IDA group’s mean score for the HHI was 28.18 (SD = 24.25, Median = 20.00, IQR = 30.5) and the control group’s mean score was 24.18 (SD = 23.61, Median = 19.00, IQR = 36.5).

#### 3.3.3. Hours of Hearing Aid Usage

The mean hours of hearing aid usage for the IDA group was 4.27 (SD = 4.42, Median = 3.46, IQR = 4.90), while for the participants of the control group, it was 3.91 (SD = 3.50, Median = 3.00, IQR = 5.63). A Mann-Whitney test was performed to compare the usage of the hearing aids by the participants of the IDA group and the participants of the control group. The test revealed no statistically significant difference between the two groups regarding the time of usage of their hearing aids, Mann-Whitney U = 133, *p* value = 0.95, n1 = 18, n2 = 15.

#### 3.3.4. Satisfaction of Hearing Aid Usage (6 Months)

The mean satisfaction for the IDA group was 7.96 (SD = 1.40, Median = 8.00, IQR = 2.00) and the mean satisfaction for the control group was 8.19 (SD = 1.38, Median = 8.00, IQR = 2.75) (Table 2). An unpaired t-test was conducted to compare the satisfaction of using the hearing aids (“t = 0.51, df = 37”) between the IDA group (n1 = 23) and the control group (n2 = 16). The test revealed no statistically significant difference between the two groups. Both the IDA and the control groups’ participants were almost equally satisfied by the hearing aid usage.

#### 3.3.5. 5-Point Likert Scale for the Effectiveness of the Living Well Counselling Tool at 4–6 Weeks

In total, 92% of the participants felt that through the IDA’s “Living Well” counselling tool they had the opportunity to discuss situations that were important for them to communicate effectively, how to handle communication in those situations and what communication strategies they were using until their “Living Well” counselling. A total of 80% of the participants found new ways of using the communication strategies in complex communication situations through the counselling tool, and 88% of them felt that they were helped to set goals for the handling of the communication strategies they had learned via the “Living Well” counselling tool (Table 4).

## 4. Discussion

Several of the IDA Institute’s tools have been used in clinical settings and are proven to facilitate the counselling between audiologists and patients [28]. The “Living Well” counselling tool, which was selected for our study, is a tool that follows the rules of person-centred counselling. It has been found that when person-centred counselling is being used, the participants are more likely to follow their counsellor’s advice and thus have the maximum benefit from their rehabilitation program [29].

### 4.1. Hearing Handicap

Based on our primary hypothesis, the two groups did not differ significantly in their 6-month HHI-A scores. The IDA group showed a gradual and consistent decrease in the hearing handicap score whereas the control group showed an immediate decrease from the baseline to the 3-month completion and then an increase between the 3-month and the 6-month completion of the questionnaire (Table 2). The reduction of the handicap score is indicative of the benefit a person receives from the amplification, so it is obvious that both groups benefitted from their hearing aids [30]. Nevertheless, the consistency in improvement of hearing handicap that is seen in the IDA group indicates that IDA’s “Living Well” may be a useful tool in obtaining long-term success. On the other hand, the control group showed a slightly higher score at the 6-month completion of the questionnaire than the 3-month completion but still much lower than the baseline, which could indicate that some sort of psychosocial adaptation may take place after exposure to a variety of listening conditions, and the patient faces some of the limitations the hearing aid technology naturally has but still feels the benefit over the unaided hearing condition [31]. This fact shows the significance of monitoring patients at least for 3 months after fitting while the problems which may arise can be resolved through counselling and/or hearing aid modification [31].

Taking into account the fact that the hearing aid fitting procedure was exactly the same for both groups in our study and was conducted by the same audiologist, the only difference would be the extra communication counselling the IDA group received. Our findings are aligned with prior studies [32,33,34,35]. Newman et al. (1993) compared the self-perceived hearing aid handicap (HHI-E) before the hearing aid fitting and 3 and 6 months after that in patients who received counselling for their hearing aids and communication counselling (cases) and patients who received only hearing aid counselling (controls) [32]. Their findings show the same trend as the control group in our study, declining rapidly from the baseline (M = 50.47, SD = 22.98) to 3 months (M = 24.18, SD = 23.61) and then increasing between the period of 3 and 6 months (M = 29.67, SD = 20.49). Similar were the results in another study conducted by Taylor (1993). That study assessed changes in the elderly subjects’ self-perceived handicap and their audiometric measurements by analysing data at pre-fitting and at 3-week, 3-month, 6-month and 1-year time intervals. After a high decrease at 3 weeks of hearing aid use, the subjects’ total HHIE scores were significantly higher at 3 months than at 3 weeks, and then they were declining again at 6 months and 1 year [34].

### 4.2. Coping Strategies

Regarding the CPHI, at six months after the hearing aid fittings and the communication counselling, the IDA group scored better in the scale of maladaptive behaviours and non-verbal strategies, the attitude of others, and the acceptance of loss, anger, discouragement and withdrawal, although again this difference was not statistically significant. In a canonical analysis, which was performed by Dermorest and Erdman (1989), it was found that the maladaptive behaviours are the most strongly related to communication effectiveness. Less use of maladaptive behaviours leads to more effective communication [10]. In our study, it is obvious that the counselling the IDA group’s participants received had an effect on their effective communication with others, whereas we could not see the same effect with the control group’s participants. This shows that the IDA’s “Living Well” counselling tool may be an effective counselling tool for first-time users of hearing aids regarding their communication coping strategies. On the other hand, the control group scored better on the scale of verbal strategies but without statistical significance (IDA group: M = 2.83, Control group: M = 3.84). The use of verbal strategies can keep to a minimum the hearing difficulties someone faces, but as it was shown in the above-mentioned canonical analysis, the verbal and non-verbal strategies are not strongly associated with the communication effectiveness someone reports [10,36]. However, this is not the only way to interpret the low correlations. The usage of verbal and non-verbal strategies may take place even if it is not mentioned or if the person does not consider communication to be effective [10]. Needless to say, this fact arises from the lack of awareness of what effective communication is and when it is present.

Another way of interpreting the CPHI results is by factors, and, as we can see from our results, the IDA group has 30.67% higher scores in the total of the scales in factor 1 (Adjustment) of the CPHI compared to the control group. As it is stated by Demorest and Erdman (1989), people who score low on the Adjustment factor are reporting negative reactions in three domains: their own feelings and attitudes, their communication strategies and the attitudes and behaviours of those with whom they interact [37]. The scores of the control group are in compliance with these negative reactions due to low scores on the Adjustment factor. Contrary to the control group, the IDA group had high scores on the Adjustment factor which shows a good adjustment.

Gomez et al. in 2001 used a model to find those variables that contribute to the use of adaptive or maladaptive strategies. Through their model, they found that people with hearing loss who use more maladaptive strategies are poorly adjusted to their hearing loss and are not receiving the social support they are expecting [16].

The IDA group also showed better scores on the scale of withdrawal and reported less anger, annoyance, and discouragement than the control group’s participants, which also showed that they communicate effectively in difficult communication situations with less sense of isolation and withdrawal from social interactions [10,27]. It may be possible that the discussion about the hearing problems, the communication strategies they can use and the hearing aid itself were beneficial for the IDA group regarding the feelings of anger due to hearing loss [36].

### 4.3. Hours of Hearing Aid Usage

The hours of hearing aid usage is another outcome to assess one’s satisfaction. In our study, the hours of usage were calculated through the data logging of the participants’ hearing aids. The IDA group used their hearing aids for 4.27 h/day on average, while the participants of the control group used them for 3.91/day on average. It has been shown in several studies that individuals who are negative regarding hearing amplification, those who feel less impacted by the hearing loss and those who believe that hearing aids may stigmatize them, use their hearing aids less [38]. In a recent study published in 2022, the researchers focused on evaluating the non-auditory measures like personality and attitude towards hearing loss on hearing aid outcome measures [39]. The average hours per day were 4.34 (SD = 2). Their results showed that participants who were aware and better accepted their hearing loss used their hearing aids more than 3.5 h/day, in comparison with those participants whose acceptance of their hearing problems was low (1.5 to 3 h/day).

### 4.4. Self-Reported Satisfaction of Hearing Aids

Clinicians tend to correlate the frequent use of hearing aids with the satisfaction of the hearing aid usage individuals may have. A study performed by Laplante-Lévesque et al. (2012) revealed that there were participants who used their hearing aids on specific occasions but still were very satisfied with them, and there were other participants who wore them the whole day and were dissatisfied [40]. It has been proven that participants who report high satisfaction using hearing aids have positive attitudes and through the daily use of their hearing aids, they may improve their everyday functioning [41].

In our study, the mean satisfaction of the IDA group’s participants regarding hearing aid usage was 7.96 (SD = 1.40) and of the control group’s participants was 8.19 (SD = 1.38) on a 0–10 Likert scale. These results are aligned with prior studies. The MarkeTrak (2022) survey, which assessed the hearing aid benefit and satisfaction of hearing aid users, revealed that the overall satisfaction across a variety of listening situations was 78% [42].

### 4.5. Generalizability of Results

Although there was no significant difference in the primary and secondary outcomes between our groups, our findings support that communication counselling could be a part of the standard hearing aid fitting procedure, especially in first-time users of hearing aids. The IDA’s “Living Well” tool was proven to be well accepted and was considered useful by the IDA group in our study. Our findings align with Scarinci et al.′s (2022) pilot study. This may imply that the “Living Well” tool is adaptable to an individuals’ heterogeneity and can be used in standard audiological care [43].

### 4.6. Limitations

One of the limitations of the present study was the high drop-out rate from the control group, which made the two groups unequal. While our initial sample consisted of 26 participants per group, the analysis included 18 people in the control group. This fact needs to be taken into account when interpreting our results. Another fact that should be taken into account when interpreting our findings is that apart from their overall satisfaction with the hearing aid experience, the two groups differed on the hours of daily hearing aid usage. This difference may be an independent factor itself affecting satisfaction whether counselling was provided or not. Moreover, this study relied mainly on self-reported data provided by the participants. While self-report measures are commonly used in research, they are subject to recall bias and may not always accurately reflect the participants’ actual behaviours or experiences.

## 5. Conclusions

Although our primary hypothesis was not confirmed, the IDA group’s participants found the “Living Well” counselling tool helpful and effective. The decrease in their hearing handicap was consistent and clinically significant 6 months after the hearing aid fitting. Use of this counselling tool with first-time hearing aid users seems to be beneficial and should be taken into account by clinicians.

## Figures and Tables

**Table 1:** The baseline demographics and clinical characteristics of the participants.

	IDA	CONTROL
Age (yrs)	Minimum: 20–Maximum: 90 Range: 70 M = 60.83 (SD = 14.83)	Minimum: 28–Maximum: 86 Range: 58 M = 60.10 (SD = 14.68)
Sex (No)	Female: 16 Male: 14	Female: 19 Male: 11
Side of Hearing Loss (No)	Bilateral: 20 Unilateral: 10	Bilateral: 17 Unilateral: 13
PTA(0.25–8 KHz, including 3 and 6 KHz)	R: M = 52.96 dB HL (SD = 9.44) L: M = 52.42 dB HL (SD = 11.35)	R: M = 48.04 dB HL (SD = 9.23) L: M = 52.29 dB HL (SD = 13.22)
SRT (Speech Reception Threshold)	R: M = 55.04 dB HL (SD = 16.47) L: M = 52.25 dB HL (SD = 15.88)	R: M = 54.11 dB HL (SD = 10.82) L: M = 57.11 dB HL (SD = 19.66)
WRS (Word Recognition Score)	R: M = 85.52% (SD = 16.27) L: M = 82.24% (SD = 16,67)	R: M = 87.81% (SD = 18.73) L: M = 90.06% (SD = 11.35)
MoCA	M = 24.33 (SD = 3.47)	M = 23.60 (SD = 3.19)
Years with Hearing Loss	M = 11.35 (SD = 9.39)	M = 8.35 (SD = 9.70)
HHI/A-E Baseline	M = 42.62 (SD = 25.97)	M = 50.47 (SD = 22.98)
CPHI Baseline	More “hospitable” communication environments and easier acceptance of the hearing impairment for the IDA Group	

**Table 2:** Outcome results for IDA and control groups.

Outcomes	IDA Group	Control Group	*p* Values
HHI 6 months	M = 24.92, SD = 20.84	M = 29.67, SD = 20.49	0.29
HHI 3 months	M = 28.18, SD = 24.25	M = 24.18, SD = 23.61	0.59
Hours of Hearing Aid Usage 6 months	M = 4.27, SD = 4.42	M = 3.91, SD = 3.50	0.95
Satisfaction of Hearing Aid Usage	M = 7.96, SD = 1.40	M = 8.19, SD = 1.38	0.61

The questionnaires results for the IDA and the control groups. The mean and standard deviation for each group and each questionnaire.

**Table 3:** Communication Profile for the Hearing Impaired (6 months completion).

CPHI Scales	IDA Group	Control Group	*p* Values
Social	M = 3.37, SD = 1.10	M = 3.69, SD = 0.81	0.31
Work	M = 3.40, SD = 1.11	M = 3.83, SD = 0.72	0.16
Home	M = 3.62, SD = 1.04	M = 3.87, SD = 0.74	0.37
Average	M = 3.65, SD = 1.06	M = 3.45, SD = 0.86	0.50
Adverse	M = 3.23, SD = 1.13	M = 3.18, SD = 0.70	0.88
Problem Awareness	M = 2.57, SD = 0.86	M = 3.52, SD = 0.84	0.0007
Need for Communication *	M = 3.20, SD = 1.10	M = 3.10, SD = 1.13	0.78
Physical Characteristics *	M = 2.79, SD = 1.09	M = 2.39, SD = 0.97	0.20
Attitudes of Others *	M = 1.90, SD = 0.71	M = 2.61, SD = 0.85	0.0045
Behaviours of Others *	M = 2.03, SD = 0.59	M = 2.17, SD = 0.85	0.50
Maladaptive Behaviours *	M = 1.76, SD = 0.53	M = 3.26, SD = 0.69	<0.0001
Verbal Strategies	M = 2.83, SD = 1.07	M = 3.84, SD = 0.90	0.0020
Nonverbal Strategies	M = 3.29, SD = 1.23	M = 2.31, SD = 0.91	0.0067
Self-acceptance *	M = 1.87, SD = 0.73	M = 2.20, SD = 0.95	0.22
Acceptance of Loss *	M = 1.79, SD = 0.65	M = 2.34, SD = 0.83	0.0175
Anger *	M = 1.96, SD = 0.73	M = 2.93, SD = 0.92	0.0003
Displacement of Responsibility *	M = 2.37, SD = 1.00	M = 2.88, SD = 0.75	0.07
Exaggeration of Responsibility *	M = 2.37, SD = 0.86	M = 2.58, SD = 0.92	0.44
Discouragement *	M = 1.91, SD = 0.74	M = 2.63, SD = 0.87	0.0031
Stress *	M = 2.12, SD = 0.81	M = 2.51, SD = 0.90	0.14
Withdrawal *	M = 2.01, SD = 0.77	M = 2.63, SD = 0.89	0.0184
Denial	M = 2.14, SD = 0.84	M = 2.63, SD = 0.89	0.07

* reversed scoring;The higher score indicates a higher level of difficulty. The CPHI scores for the IDA group and the control group. The mean and standard deviation for each scale.

**Table 4:** The satisfaction for the “Living Well” counselling tool (proportion of participants who scored over 3).

**Living Well**	**Baseline**	**4–6 Weeks**
The IDA’s “Living Well” counselling tool gave me the opportunity to discuss situations which are important for me to communicate effectively.	87%	92%
By using the IDA’s “Living Well” counselling tool, I could discuss ways with which to handle a communication situation which is important for me.	90%	92%
The IDA’s “Living Well” counselling tool gave me the opportunity to recognize the communication strategies I used until now.	80%	92%
Through the IDA’s “Living Well” counselling tool, new ways of using the communication strategies in complex communication situations were presented to me.	73%	80%
The IDA’s “Living Well” counselling tool helped me to set goals for the handling of the communication strategies.	80%	88%

The proportion of the IDA group’s participants who scored over 3 on a 1–5 Likert scale about the satisfaction of the “Living Well” tool at the baseline and 4–6 weeks after the initial appointment.

## Data Availability

The original contributions presented in the study are included in the article, further inquiries can be directed to the corresponding author.
